# Abnormal Nailfold Capillaries in Patients after Hand Transplantation

**DOI:** 10.3390/jcm9113422

**Published:** 2020-10-25

**Authors:** Dorota Sikorska, Włodzimierz Samborski, Dorota Kamińska, Mariusz Kusztal, Jerzy Jabłecki, Kacper Nijakowski, Andrzej Oko, Marek Karczewski, Katarzyna Korybalska, Janusz Witowski

**Affiliations:** 1 Department of Rheumatology and Rehabilitation, Poznan University of Medical Sciences, 61-545 Poznan, Poland; samborskiw@o2.pl; 2 Department of Nephrology and Transplantation Medicine, Wroclaw Medical University, 50-556 Wroclaw, Poland; dorotakaminska@interia.pl (D.K.); mariok@o2.pl (M.K.); 3 Subdepartment of Replantation of Limbs, St. Hedwig of Silesia Hospital, ul Prusicka 53, 55-100 Trzebnica, Poland; jerzy.jablecki@interia.pl; 4 State Higher Medical Professional School, ul Katowicka 68, 45-060 Opole, Poland; 5 Department of Conservative Dentistry and Endodontics, Poznan University of Medical Sciences, 60-812 Poznan, Poland; kacpernijakowski@ump.edu.pl; 6 Department of Nephrology, Transplantology and Internal Medicine, Poznan University of Medical Sciences, 60-355 Poznan, Poland; aoko@ump.edu.pl; 7 Department of General and Transplant Surgery, Poznan University of Medical Sciences, 60-355 Poznan, Poland; drkarczewski@gmail.com; 8 Department of Pathophysiology, Poznan University of Medical Sciences, 60-806 Poznan, Poland; koryb@ump.edu.pl (K.K.); jwitow@ump.edu.pl (J.W.)

**Keywords:** hand transplantation, nailfold capillaroscopy, vascular endothelial growth factor, microvascular abnormalities, angiogenesis

## Abstract

Background: The development of graft vasculopathy may play a role in the long-term deterioration of hand grafts. The aim of study was to examine the patterns of the nailfold capillaries in hand transplant recipients. Methods: the study was performed on six patients who received hand transplantation. To normalize for the effect of immunosuppression an age- and sex-matched group of 12 patients with active kidney transplant was selected. As an additional control group, 12 healthy volunteers were recruited. Nailfold videocapillaroscopy was performed in all participants. Additionally, serum concentrations of vascular endothelial growth factor (VEGF) were measured. Results: Videocapillaroscopic examination of the hand allografts revealed significant abnormalities: including capillary disorganization and microhaemorrhages. The number of capillaries was reduced, the vessels were enlarged and branched. Surprisingly, similar, albeit slightly less pronounced, changes were seen in the nailfolds of healthy hands of the limb transplant recipients. In kidney transplant recipients the capillaroscopic pattern was general normal and comparable to healthy individuals. Moreover, serum concentrations of VEGF in all participants correlated with average capillary diameter in capillaroscopy. Conclusions: in hand transplant recipients advanced microvascular abnormalities are found in nailfold capillaroscopic pattern in both transplanted and own extremities connected with elevated levels of VEGF.

## 1. Introduction

The first successful hand transplantation was performed in 1998 in Lyon, France [1]. Although the lack of patient’s compliance with the immunosuppression regimen led eventually to transplant rejection in 2001, subsequent attempts of limb transplantation were a success. The adequate patient selection, advancements in surgical techniques, comprehensive postoperative rehabilitation and modern immunotherapy contributed to successful outcomes [2,3]. However, longer transplant survival rates revealed other potential problems, such as chronic graft rejection [4,5] or chronic kidney disease and cardiovascular complications—which can cause changes in the microcirculation [6]. As hand transplantation is not a life-saving procedure, the discussions on advantages and disadvantages of the procedure have intensified [2,3]. Currently, there is a published record of more than 120 upper limbs transplanted into 74 patients [3,7]. The Polish experience amounts to eight upper extremity transplantations in seven patients [8,9]. 

It has been estimated that 85–90% of hand transplant recipients experienced at least 1 episode of acute rejection, which could usually be controlled when promptly treated [2,10]. However, as in solid-organ transplants, hand allografts can also undergo chronic rejection with serious clinical consequences [4,5]. The upper limb allotransplant is composed of a number of different tissues with different immunogenicity. Of these, the skin appears to be the most immunogenic and becomes the main target for rejection. As the number of hand transplantations is small and the follow-up periods are relatively short, there is little data on chronic rejection of hand allografts. It appears, however, that similarly to solid-organ transplants, the development of graft vasculopathy may play a role in the long-term deterioration of hand grafts. The mechanisms underlying hand graft vasculopathy are not clear. They may include both immunological and non-immunological (mechanical, cytotoxic and thermal) insults that lead to endothelial cell damage and vessel remodeling [7,11,12,13]. 

An early diagnosis of graft vasculopathy may help to modify immunosuppression in time [5]. In solid-organ transplantation, some laboratory tests may herald the developing rejection [14]. It has been suggested that serum levels of vascular endothelial growth factor (VEGF) and other angiogenic factors may serve as diagnostic markers of allograft vasculopathy in cardiac transplant recipients [15,16,17]. Little is known on whether such associations exist in hand transplant recipients. Rejection of hand allografts are apparent predominantly in the skin vessels and can be suspected when patients develop skin changes, cutaneous ulcerations, necrosis, or sclerosis, thinning of the fingers and the loss of nails [4,5]. Unfortunately, however, these features develop at advanced stages of rejection and are irreversible. The current methods of monitoring limb transplant recipients for chronic rejection cannot detect graft vasculopathy at early stages [18]. Probably vascular dysfunction is present in all hand transplant patients, despite apparently normal vascular images by conventional methods and unremarkable histology of skin biopsies [5]. That is why new methods for early diagnosis of vasculopathy in hand transplant patients are needed. 

Nailfold capillaroscopy appears to be a promising method for detecting vasculopathy in hand grafts [19,20]. It is a simple, non-invasive, easily repeatable and elegant tool to study microvascular architecture [21]. Nailfold capillaroscopy enables early detection and qualitative description of microvascular abnormalities in the skin. The typical pattern of the nailfold microvasculature is characterized by a uniform distribution of hairpin-shaped capillaries. While tortuous and criss-crossing capillaries may also be observed in the normal vasculature, disorganized or branching capillaries, avascular areas and microhaemorrhages are all considered incorrect. The normal capillary density ranges from 9 to 12 capillaries per linear millimeter, and the normal capillary diameter is <20 µm. The capillaries ≥20 and ≥50 µm are defined as ectasic and giant, respectively [22,23,24]. Capillaroscopy is a well-recognized tool for evaluating microvasculature in connective tissue diseases, especially in systemic sclerosis [19,21]. An attempt was also made to use it for assessing circulation in replanted severed fingers [25]. 

The aim of our study was to examine the patterns of the nailfold capillaries in patients with transplanted upper extremities and relate them to the levels of serum VEGF. 

## 2. Experimental Section

The study was performed on six patients who received forearm (*n* = 5) or arm (*n* = 1) transplantation in St. Hedwig of Silesia Hospital, Trzebnica, Poland. The patients lost their limbs as a result of trauma and were in a good health before transplantation. Immunosuppressive treatment included basiliximab in induction and triple drug therapy based on tacrolimus, mycophenolate mofetil, and prednisone at doses presented earlier [26]. After transplantation, the patients were routinely examined for allograft condition. All of hand transplant recipients experienced at least 1 episode of acute rejection (from 1 to 6), which was controlled. The patients were examined 2 to 12 years (mean 9 ± 5 years) after transplantation. They were all in a stable clinical condition with no evidence of acute transplant rejection. To normalize for the effect of immunosuppression the age- and sex-matched group of 12 patients with an active kidney transplant was selected. These patients suffered from a primary kidney disease without co-morbidities and had a good function of the transplanted kidney. Both groups received the same triple immunosuppression (tacrolimus + mycophenolate mofetil + prednisone) at standard doses [26] for a comparable period of time. As an additional control group, 12 healthy volunteers of a similar age (mean 35 ± 5 years) were recruited from the general population. The study protocol conformed to the ethical guidelines of the 1975 Declaration of Helsinki and it was approved by the Poznan University Ethics Committee (No. 16/18). All experiments were performed in accordance with relevant named guidelines and regulations. No organs/tissues were procured from prisoners. Written informed consent was obtained from each participant.

Nailfold videocapillaroscopy was performed by the same experienced operator (DS) using the CapillaryScope 200 MEDL4N microscope (Dino-Lite; Europe, Almere, The Netherlands). Nailfold capillaroscopy is a simple, non-invasive, easily repeatable and elegant tool to study microvascular architecture—the cost of purchasing a videocapillaroscope is about EUR 1000, and individual examinations are basically free (they only require the researcher’s time). The examination took place at room temperature, after 20 min of rest and involved both hands and all fingers (excluding thumbs). A global examination of the entire nailfold area and microvascular network was performed under low (50×) magnification. Then, 3 pictures at high (200×) magnification were taken from each finger (12 pictures for one hand) to assess the (1) morphology, (2) density and (3) diameter of the capillaries. All capillaries captured were assessed and then the average of all measurements was calculated [21]. The capillary density was estimated by counting the number of capillaries per linear millimeter. A patient was defined as having abnormal capillaries if one type of abnormality was present in at least two fingers. 

Samples of serum were collected by routine methods at the time of nailfold videocapillaroscopy. Serum concentrations of VEGF were measured using the DuoSet^®^ Immunoassay Kit (R&D Systems; Bio-Techne, Poland) with estimated sensitivity of 13 pg/mL. The immunoassay was performed as per manufacturer’s instructions. 

Statistical analyses were performed using the Statistica 13.0 software (StatSoft Polska, Krakow, Poland). Since the number of patients was too small to ascertain normality of the data distribution, non-parametric statistics were applied and the data were presented as medians (and interquartile ranges) or as percentages, as appropriate. The data were analyzed with the Kruskal–Wallis or Mann–Whitney tests, as required. Categorized data were analyzed with the χ2 test. The relationship between variables was analyzed with the Spearman’s rank correlation coefficient. The differences were considered significant at *p* < 0.05. 

## 3. Results

There were no differences in basic characteristics between hand- and kidney transplant recipients analyzed (Table 1). 

**Figure 1:**
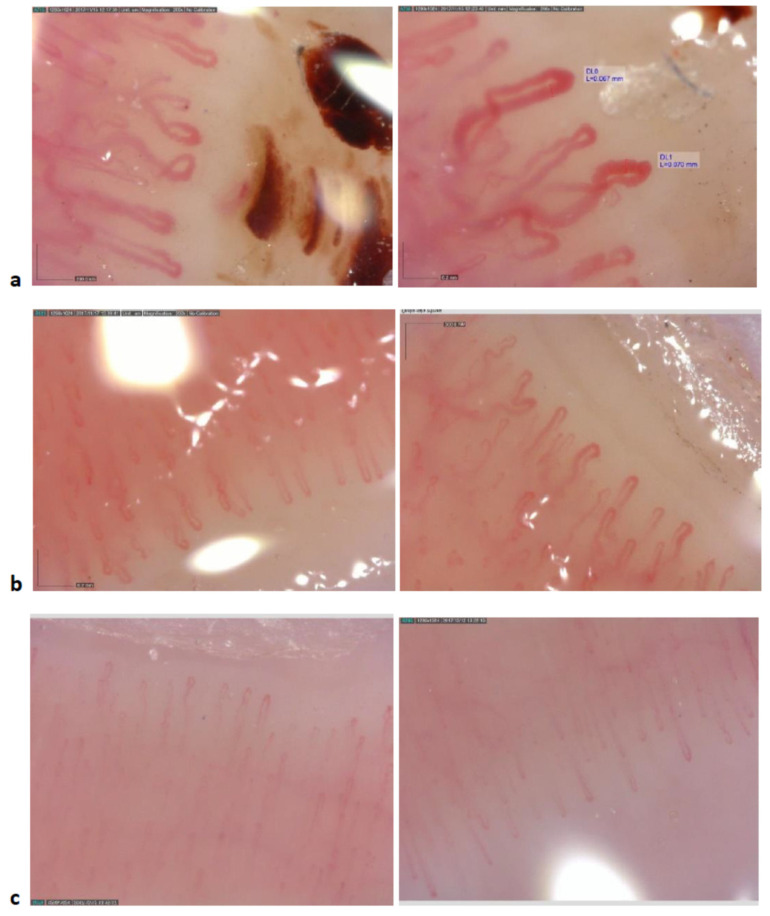
The nailfold capillaroscopic pattern: (**a**) hand allografts; (**b**) healthy hands in patients after limb transplantation; (**c**) hands in patients after kidney transplantation.

**Figure 2:**
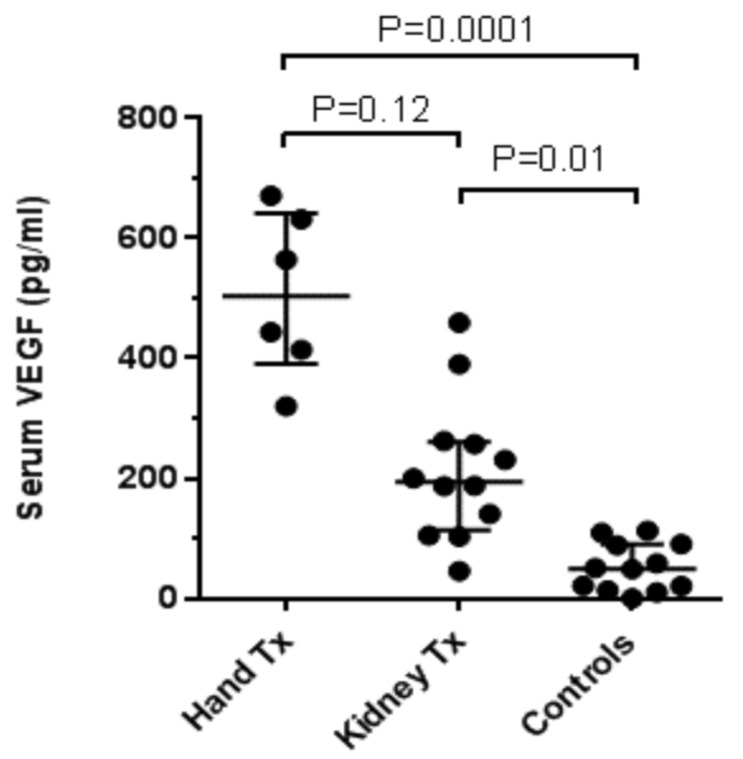
Serum vascular endothelial growth factor (VEGF) levels in subgroup.

Videocapillaroscopic examination of the hand allografts revealed significant abnormalities in the microvasculature, including capillary disorganization and the presence of microhaemorrhages. The number of capillaries was reduced, the vessels were enlarged and branched (Figure 1a and Table 2). Surprisingly, very similar, albeit slightly less pronounced, changes were seen in the nail folds of healthy hands of the limb transplant recipients (Figure 1b and Figure 2). By contrast, in kidney transplant recipients the capillaroscopic appearance was generally normal (Figure 1c) and resembled that seen in healthy individuals (Table 2). 

Although the capillary density in the hand allografts was reduced in all patients, there was no statistically significant difference between the average number of capillaries between hands (graft vs. healthy) and between groups—probably due to the small number of patients in the groups. 

Interestingly, the maximal diameter of capillaries in the hand allografts was significantly greater than that found in healthy hands (*p* = 0.013), which, in turn, was greater than that in kidney transplant patients (*p* = 0.010). Consequently, the average diameter of capillaries in patients after limb transplantation was significantly greater than that in the kidney transplant group (*p* = 0.001). Finally, the capillary diameters in kidney transplant recipients did not differ significantly from those recorded in healthy individuals.

**Figure 3:**
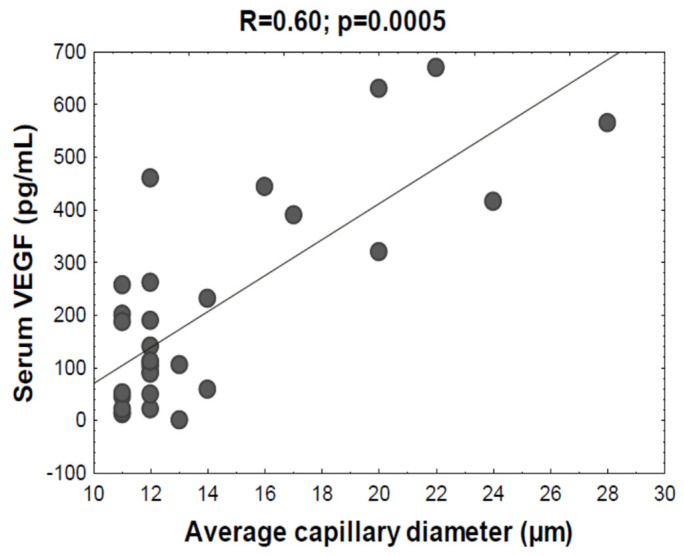
Correlation between serum VEGF levels and average capillary diameter.

Serum concentrations of VEGF in either hand or kidney transplant patients were significantly higher than those in healthy controls (Figure 2). Furthermore, the levels of VEGF in hand transplant patients were higher compared with kidney transplant patients (although the Dunn’s test for multiple comparisons failed to reach formal significance at *p* = 0.12, the direct comparison of the two groups using the Mann–Whitney test pointed to a significant difference at *p* = 0.0013). Finally, serum concentrations of VEGF in all participants correlated with average capillary diameter recorded during capillaroscopy (R = 0.60; *p* = 0.0005) (Figure 3).

## 4. Discussion

The main finding of our study is that hand allograft recipients display significant abnormalities in the nailfold microvasculature as assessed by capillaroscopy. These abnormalities resemble those seen in patients with active systemic sclerosis [27]. Microangiopathy in systemic sclerosis progresses gradually from perivascular inflammation, through excessive accumulation of extracellular matrix to capillary damage followed by pathological neoangiogenesis. These processes are believed to be largely driven by tissue hypoxia, which activates transcription factor HIF (hypoxia-inducible factor) that can subsequently induce VEGF [28,29]. VEGF, in turn, is the main inducer of pathological angiogenesis [30]. Indeed, we have observed both elevated VEGF and significant capillary abnormalities in the nailfolds of patients with hand allografts. 

Surprisingly, we have found that all limb transplant recipients also had significant capillary abnormalities in the nailfolds of healthy hands. Such changes were not seen in kidney transplant recipients. Having detected high VEGF levels in hand transplant patients, one can speculate that chronic ischemia within the allograft promotes the release of angiogenic factors into systemic circulation, which eventually results in abnormal neoagiogenesis in healthy hands [31]. In addition, prolonged ischemia in transplanted limbs can lead to the release of molecules known as damage-associated molecular patterns (e.g., reactive oxygen species, heat shock proteins), which can initiate and drive chronic inflammatory response [7]. It was shown that hand transplant recipients exhibit persistent immune activation with rejection-related gene expression pattern [32]. In this respect, several key pro-inflammmatory mediators can induce VEGF [33,34,35]. The fact that vascuopathy appears to be more pronounced in hand transplant patients than in solid-organ recipients may be because the skin is a very immunogenic tissue and the target for rejection-associated inflammation [36]. There are no studies on nailfold capillaroscopic patterns in patients after transplantations, but high levels of VEGF have been demonstrated to associate strongly with vasculopathy seen during the rejection of cardiac transplants [15,16,17]. Moreover, it has been observed that serum VEGF correlated with the presence of nailfold capillary abnormalities in patients with autoimmune diseases [37,38]. In addition, it has been proposed that nailfold capillaroscopic patterns and VEGF levels may serve as biomarkers of interstitial lung involvement in systemic sclerosis [33,34,35]. It could be argued that changes seen in the capillaroscopy in transplanted patients are related to the ongoing immunosuppression and metabolic complications that it causes [6,39,40]. However, this is probably not a decisive factor as in kidney transplant patients on the same immunosuppressive regimen the degree of capillary abnormalities and VEGF levels were significantly less. 

The clinical significance of the observed changes in microcirculation is also ambiguous. Reduced microcirculation blood flow may probably cause cold intolerance following digit replantation and reflex sympathetic dystrophy [41]. However, in our research the changes in the videocapillaroscopic pattern did not translate into the function of the transplanted limb. All the patients in the study group experienced at least one episode of acute rejection (from 1 to 6), but no relationship was observed between the number of acute rejection episodes and the capillaroscopic pattern. At the same time, the most advanced changes in microcirculation were observed in the two longest post-transplant patients (9 and 11 years after transplantation). Due to the small size of the study group, it is difficult to draw clear conclusions. 

The main limitation of our study is the small number of patients studied. An additional, but important limitation of the study is the only one-time assessment and the lack of capillaroscopic pattern of the healthy limb before transplantation, which means that we do not have reference to the baseline state or assessment of changes over time. As such they need to be confirmed by other studies, which may require international collaboration since the number of patients after limb transplantation is generally low (here, we have examined all patients with unilateral hand allografts in our country). To the best of our knowledge, there are no reports in the literature on the association between limb transplantation and changes in nailfold capillaries, so far. Our observations are obviously preliminary and require further research and confirmation in subsequent studies on a larger group of patients. 

## 5. Conclusions

In hand transplant recipients advanced microvascular abnormalities are found in nailfold capillaroscopic pattern in both transplanted and own extremities connected with elevated levels of VEGF. Nailfold capillaroscopy may be a useful method for assessing microcirculation in hand allografts.

## Figures and Tables

**Table 1:** Patients’ characteristics.

	Patients after Hand Transplantation (*n* = 6)	Patients after Kidney Transplantation (*n* = 12)	*p*-Value
Men, *n* (%)	5 (83%)	10 (83%)	1.000
Age, years	38 (33–45)	47 (43–63)	0.089
Cause of transplantation, *n* (%)	Trauma: 6 (100%)	Primary glomerulonephritis: 6 (50%) Polycystic kidney disease: 3 (25%) Chronic tubulointerstitial nephritis: 3 (25%)	NA
Time after transplantation, years	8 (3–9)	11 (6–15)	0.188
Post-steroid diabetes mellitus, *n* (%)	1 (17%)	2 (17%)	1.000
Hypertension, *n* (%)	1 (17%)	7 (58%)	0.093

Data are presented as a median (interquartile range) or *n* (%); NA—not applicable.

**Table 2:** Patients’ characteristics.

	Patients after Hand Transplantation (*n* = 6) -Hand Allografts	Patients after Hand Transplantation (*n* = 6) -Healthy Hands	Patients after Kidney Transplantation(*n* = 12) -Both Hands	Healthy Control Group(*n* = 12) -Both Hands
capillary disorganization, *n* (%)	2 (33%)	0 (0%)	0 (0%)	0 (0%)
avascular areas, *n* (%)	0 (0%)	0 (0%)	0 (0%)	0 (0%)
capillary density (per mm)	6 (5–6)	7 (7–8)	8 (8–9)	8 (8–9)
hemorrhages, *n* (%)	3 (50%)	1 (17%)	1 (8%)	0 (0%)
tortuous capillaries, *n* (%)	6 (100%)	6 (100%)	7 (58%)	5 (42%)
branching capillaries, *n* (%)	4 (67%)	3 (50%)	1 (8%)	0 (0%)
ectasic capillaries, *n* (%)	6 (100%)	6 (100%)	5 (42%)	4 (33%)
giant capillaries, *n* (%)	3 (50%)	0 (0%)	0 (0%)	0 (0%)
maximal diameter of capillaries (µm)	48 (42–56)	30 (27–36)	20 (18–26)	19 (19–25)
average diameter of capillaries (µm)	29 (26–32)	21 (20–24)	12 (11–12)	12 (11–13)

Data presented as: median (interquartile range) or *n* (%).

## References

[1] Dubernard J.M., Owen E., Herzberg G., Lanzetta M., Martin X., Kapila H., Dawahra M., Hakim N.S. (1999). Human hand allograft: Report on first 6 months. Lancet.

[2] Petruzzo P., Lanzetta M., Dubernard J.M., Landin L., Cavadas P., Margreiter R., Schneeberger S., Breidenbach W., Kaufman C., Jablecki J. (2010). The International Registry on Hand and Composite Tissue Transplantation. Transplantation.

[3] Shores J.T., Malek V., Lee W.P.A., Brandacher G. (2017). Outcomes after hand and upper extremity transplantation. J. Mater. Sci. Mater. Med..

[4] Kanitakis J., Petruzzo P., Badet L., Gazarian A., Thaunat O., Testelin S., Devauchelle B., Dubernard J.M., Morelon E. (2016). Chronic Rejection in Human Vascularized Composite Allotransplantation (Hand and Face Recipients): An Update. Transplantation.

[5] Kaufman C.L., Ouseph R., Blair B., Kutz J.E., Tsai T.M., Scheker L.R., Tien H.Y., Moreno R., Ozyurekoglu T., Banegas R. (2012). Graft vasculopathy in clinical hand transplantation. Am. J. Transplant. Off. J. Am. Soc. Transplant. Am. Soc. Transpl. Surg..

[6] Boratynska M., Obremska M., Malecki R., Gacka M., Magott M., Kaminska D., Banasik M., Kusztal M., Chelmonski A., Jablecki J. (2014). Impact of immunosuppressive treatment on the cardiovascular system in patients after hand transplantation. Transplant. Proc..

[7] Kollar B., Kamat P., Klein H.J., Waldner M., Schweizer R., Plock J.A. (2019). The Significance of Vascular Alterations in Acute and Chronic Rejection for Vascularized Composite Allotransplantation. J. Vasc. Res..

[8] Jablecki J., Kaczmarzyk L., Domanasiewicz A., Chelmonski A., Paruzel M., Elsaftawy A., Syrko M., Kaczmarzyk J. (2012). Result of arm-level upper-limb transplantation in two recipients at 19- and 30-month follow-up. Ann. Transplant..

[9] Jablecki J. (2011). World experience after more than a decade of clinical hand transplantation: Update on the Polish program. Hand Clin..

[10] Hein R.E., Ruch D.S., Klifto C.S., Leversedge F.J., Mithani S.K., Pidgeon T.S., Richard M.J., Cendales L.C. (2019). Hand transplantation in the United States: A review of the Organ Procurement and Transplantation Network/United Network for Organ Sharing Database. Am. J. Transplant. Off. J. Am. Soc. Transplant. Am. Soc. Transpl. Surg..

[11] Etra J.W., Raimondi G., Brandacher G. (2018). Mechanisms of rejection in vascular composite allotransplantation. Curr. Opin. Organ Transplant..

[12] Morelon E., Petruzzo P., Kanitakis J. (2018). Chronic rejection in vascularized composite allotransplantation. Curr. Opin. Organ Transplant..

[13] Borges T.J., O’Malley J.T., Wo L., Murakami N., Smith B., Azzi J., Tripathi S., Lane J.D., Bueno E.M., Clark R.A. (2016). Codominant Role of Interferon-gamma- and Interleukin-17-Producing T Cells During Rejection in Full Facial Transplant Recipients. Am. J. Transplant. Off. J. Am. Soc. Transplant. Am. Soc. Transpl. Surg..

[14] Krstic D., Tomic N., Radosavljevic B., Avramovic N., Dragutinovic V., Skodric S.R., Colovic M. (2016). Biochemical Markers of Renal Function. Curr. Med. Chem..

[15] Daly K.P., Seifert M.E., Chandraker A., Zurakowski D., Nohria A., Givertz M.M., Karumanchi S.A., Briscoe D.M. (2013). VEGF-C, VEGF-A and related angiogenesis factors as biomarkers of allograft vasculopathy in cardiac transplant recipients. Am. J. Transplant. Off. J. Am. Soc. Transplant. Am. Soc. Transpl. Surg..

[16] Stehlik J., Armstrong B., Baran D.A., Bridges N.D., Chandraker A., Gordon R., De Marco T., Givertz M.M., Heroux A., Ikle D. (2019). Early immune biomarkers and intermediate-term outcomes after heart transplantation: Results of Clinical Trials in Organ Transplantation-18. Am. J. Transplant. Off. J. Am. Soc. Transplant. Am. Soc. Transpl. Surg..

[17] Watanabe K., Karimpour-Fard A., Michael A., Miyamoto S.D., Nakano S.J. (2018). Elevated serum vascular endothelial growth factor and development of cardiac allograft vasculopathy in children. J. Heart Lung Transplant. Off. Publ. Int. Soc. Heart Transplant..

[18] Kanitakis J., Karayannopoulou G., Lanzetta M., Petruzzo P. (2014). Graft vasculopathy in the skin of a human hand allograft: Implications for diagnosis of rejection of vascularized composite allografts. J. Heart Lung Transplant. Off. Publ. Int. Soc. Heart Transplant..

[19] Ingegnoli F., Smith V., Sulli A., Cutolo M. (2018). Capillaroscopy in Routine Diagnostics: Potentials and Limitations. Curr. Rheumatol. Rev..

[20] Klein-Weigel P.F., Sunderkotter C., Sander O. (2016). Nailfold capillaroscopy microscopy–an interdisciplinary appraisal. VASA Z. Gefasskrankh..

[21] Cutolo M., Sulli A., Smith V. (2013). How to perform and interpret capillaroscopy. Best practice & research. Clin. Rheumatol..

[22] Cutolo M., Smith V. (2013). State of the art on nailfold capillaroscopy: A reliable diagnostic tool and putative biomarker in rheumatology?. Rheumatology.

[23] Cutolo M., Pizzorni C., Secchi M.E., Sulli A. (2008). Capillaroscopy. Best Pract. Res. Clin. Rheumatol..

[24] Cutolo M., Pizzorni C., Sulli A. (2005). Capillaroscopy. Best practice & research. Clin. Rheumatol..

[25] Lu W., Wang D., Liu L., Xiong J., He Q. (2008). Nail fold capillary observation in replanted severed fingers. Microsurgery.

[26] Jablecki J., Kaczmarzyk L., Patrzalek D., Domanasiewicz A., Boratynska Z. (2009). First Polish forearm transplantation: Report after 17 months. Transplant. Proc..

[27] Ruaro B., Sulli A., Smith V., Pizzorni C., Paolino S., Alessandri E., Cutolo M. (2017). Microvascular damage evaluation in systemic sclerosis: The role of nailfold videocapillaroscopy and laser techniques. Reumatismo.

[28] Ioannou M., Pyrpasopoulou A., Simos G., Paraskeva E., Nikolaidou C., Venizelos I., Koukoulis G., Aslanidis S., Douma S. (2013). Upregulation of VEGF expression is associated with accumulation of HIF-1alpha in the skin of naive scleroderma patients. Mod. Rheumatol..

[29] Distler J.H., Jungel A., Pileckyte M., Zwerina J., Michel B.A., Gay R.E., Kowal-Bielecka O., Matucci-Cerinic M., Schett G., Marti H.H. (2007). Hypoxia-induced increase in the production of extracellular matrix proteins in systemic sclerosis. Arthritis Rheum..

[30] Nagy J.A., Dvorak A.M., Dvorak H.F. (2007). VEGF-A and the induction of pathological angiogenesis. Annu. Rev. Pathol..

[31] Tuomisto T.T., Rissanen T.T., Vajanto I., Korkeela A., Rutanen J., Yla-Herttuala S. (2004). HIF-VEGF-VEGFR-2, TNF-alpha and IGF pathways are upregulated in critical human skeletal muscle ischemia as studied with DNA array. Atherosclerosis.

[32] Kaminska D., Koscielska-Kasprzak K., Krajewska M., Chelmonski A., Jablecki J., Zabinska M., Myszka M., Banasik M., Boratynska M., Gomolkiewicz A. (2017). Immune activation- and regulation-related patterns in stable hand transplant recipients. Transpl. Int. Off. J. Eur. Soc. Organ Transpl..

[33] Zhou Y., Hou W., Xu K., Han D., Jiang C., Mou K., Li Y., Meng L., Lu S. (2015). The elevated expression of Th17-related cytokines and receptors is associated with skin lesion severity in early systemic sclerosis. Hum. Immunol..

[34] Pendergrass S.A., Hayes E., Farina G., Lemaire R., Farber H.W., Whitfield M.L., Lafyatis R. (2010). Limited systemic sclerosis patients with pulmonary arterial hypertension show biomarkers of inflammation and vascular injury. PLoS ONE.

[35] Distler O., Distler J.H., Scheid A., Acker T., Hirth A., Rethage J., Michel B.A., Gay R.E., Muller-Ladner U., Matucci-Cerinic M. (2004). Uncontrolled expression of vascular endothelial growth factor and its receptors leads to insufficient skin angiogenesis in patients with systemic sclerosis. Circul. Res..

[36] Kadono K., Gruszynski M., Azari K., Kupiec-Weglinski J.W. (2019). Vascularized composite allotransplantation versus solid organ transplantation: Innate-adaptive immune interphase. Curr. Opin. Organ Transplant..

[37] Barbulescu A.L., Vreju A.F., Buga A.M., Sandu R.E., Criveanu C., Tudorascu D.R., Gheonea I.A., Ciurea P.L. (2015). Vascular endothelial growth factor in systemic lupus erythematosus—Correlations with disease activity and nailfold capillaroscopy changes. Rom. J. Morphol. Embryol. Rev. Roum. Morphol. Embryol..

[38] De Santis M., Ceribelli A., Cavaciocchi F., Crotti C., Massarotti M., Belloli L., Marasini B., Isailovic N., Generali E., Selmi C. (2016). Nailfold videocapillaroscopy and serum VEGF levels in scleroderma are associated with internal organ involvement. Auto Immun. Highlights.

[39] Bongard O., Weimer D., Lemoine R., Bolle J.F., Leski M., Bounameaux H. (2000). Cyclosporine toxicity in renal transplant recipients detected by nailfold capillaroscopy with Na-fluorescein. Kidney Int..

[40] Triantafyllou A., Anyfanti P., Triantafyllou G., Zabulis X., Aslanidis S., Douma S. (2016). Impaired metabolic profile is a predictor of capillary rarefaction in a population of hypertensive and normotensive individuals. J. Am. Soc. Hypertens. JASH.

[41] Pollock F.E., Smith T.L., Koman L.A. (1994). Microvascular response in the rabbit ear to total body cooling: A model for study of human digits. Microsurgery.

